# Evaluation of cerebrospinal fluid proteins as potential biomarkers for early stage Parkinson’s disease diagnosis

**DOI:** 10.1371/journal.pone.0206536

**Published:** 2018-11-01

**Authors:** Marcia Cristina T. dos Santos, Dieter Scheller, Claudia Schulte, Irene R. Mesa, Peter Colman, Sarah R. Bujac, Rosie Bell, Caroline Berteau, Luis Tosar Perez, Ingolf Lachmann, Daniela Berg, Walter Maetzler, Andre Nogueira da Costa

**Affiliations:** 1 Translational Medicine, UCB Biopharma SPRL, Braine L’Alleud, Belgium; 2 Consultancy Neuropharm, Neuss, Germany; 3 Hertie Institute for Clinical Brain Research, Department of Neurodegeneration, University of Tuebingen and German Center for Neurodegenerative Diseases, Tuebingen, Germany; 4 Exploratory Statistics, Global Exploratory Development, UCB Pharma SA, Slough, United Kingdom; 5 Bioanalytical Sciences, Non Clinical Development, UCB Biopharma SPRL, Braine L’Alleud, Belgium; 6 Research and Development, Analytik Jena, Jena, Germany; 7 Department of Neurology, Christian-Albrechts-University Kiel, Kiel, Germany; Nathan S Kline Institute, UNITED STATES

## Abstract

Cerebrospinal fluid (CSF) has often been used as the source of choice for biomarker discovery with the goal to support the diagnosis of neurodegenerative diseases. For this study, we selected 15 CSF protein markers which were identified in previously published clinical investigations and proposed as potential biomarkers for PD diagnosis. We aimed at investigating and confirming their suitability for early stage diagnosis of the disease. The current study was performed in a two-fold confirmatory approach. Firstly, the CSF protein markers were analysed in confirmatory cohort I comprising 80 controls and 80 early clinical PD patients. Through univariate analysis we found significant changes of six potential biomarkers (α-syn, DJ-1, Aβ42, S100β, p-Tau and t-Tau). In order to increase robustness of the observations for potential patient differentiation, we developed–based on a machine learning approach—an algorithm which enabled identifying a panel of markers which would improve clinical diagnosis. Based on that model, a panel comprised of α-syn, S100β and UCHL1 were suggested as promising candidates. Secondly, we aimed at replicating our observations in an independent cohort (confirmatory cohort II) comprising 30 controls and 30 PD patients. The univariate analysis demonstrated Aβ42 as the only reproducible potential biomarker. Taking into account both technical and clinical aspects, these observations suggest that the large majority of the investigated CSF proteins currently proposed as potential biomarkers lack robustness and reproducibility in supporting diagnosis in the early clinical stages of PD.

## Introduction

Parkinson’s disease (PD) is a complex neurodegenerative disorder which leads to progressive impairment of motor function caused by prominent loss of dopamine-secreting neurons within the substantia nigra [1, 2]. PD diagnosis so far is based only on clinical criteria by identification of neuro-motor symptoms, the accuracy of which is limited during the first years of the disease [3]. Recent findings in the literature reveal the emergence of non-motor symptoms years before a clinical diagnosis of PD can be made [4]. Currently, it is well known that non-motor symptoms are major contributors to disease morbidity as the disease progresses, frequently receding motor symptoms and involving central and peripherical non-dopaminergic signaling [4, 5]. It is believed that during this initial phase, neurodegeneration is not extensive and severe, thus offering a perfect window for novel therapies and disease modifying treatments. However, diagnosis of early PD would require further parameters in addition to the clinical criteria.

To date, PD is still incurable; treatments available consist of drugs designed to increase dopamine levels and relieve motor symptoms but not to alter disease progression [6]. The development of improved standard of care and novel therapies, that may impede degeneration and delay PD progression, is limited by the already significant degeneration of a large number of dopaminergic neurons at the time clinical diagnosis can be established [7, 8]. The deployment of biomarkers reflecting the onset and progression of the pathology accurately may have a profound impact on diagnosis and detection of the disease. The identification of clinically applicable biomarkers for the early clinical stages of PD, therefore, will not only facilitate clinical diagnosis, but also help monitoring disease progression, enable personalized therapies and open a window for disease modifying treatments.

Currently, biomarkers for PD diagnosis have been extensively investigated, however their clinical applicability still warrants further efforts [9]. Several imaging approaches have been developed to provide in-depth information on brain structure and functioning [10]. However, these techniques are not easily employed in the clinical setting due to the high costs and the demands on infrastructure and therefore are limited when considering routine use for early diagnosis. Molecular biomarkers detectable in body fluids are considered the ideal approach to facilitate clinical diagnosis. Concerning neurodegenerative diseases, CSF is the preferred biofluid for biomarker investigations due to its supposed surrogacy to the brain [11]. Despite the popularity of CSF for biomarker research in PD, validated biomarkers applicable in a clinical setting are still limited. Several studies have highlighted altered levels of α-synuclein (α-syn), DJ-1, amyloid beta (Aβ) and Tau proteins in late stage, symptomatically identified PD patients compared with controls [12–17]. Nevertheless, these findings are not consistently observed in different studies [18].

The current study was designed to explore the suitability of a panel of 15 CSF protein markers which had been proposed as potential biomarkers based on corresponding discovery studies performed in late stage PD patients, with clinical diagnosis obtained between 74 to 136 months of already existing symptoms. This study aimed at exploring those protein markers in order to confirm their potential as biomarkers applicable to early clinical PD patients that were diagnosed during a symptomatic period of up to 36 months after first signs of the disease.

Our approach closely mimicked suggestions for the performance of a reliable biomarker study in the Alzheimer disease (AD) field. As suggested by a consensus report for biomarkers in AD, a biomarker should make use of a fundamental feature of neuropathology and be investigated in neuropathologically confirmed cases; furthermore, it should have a high diagnostic sensitivity and specificity (of >80%) for distinguishing other dementias. The potential biomarker should be investigated by at least two independent studies, in our case consisting of at least two separate cohorts [19, 20].

In this study, the confirmation was conducted in a two-step approach: a first confirmatory phase I (employing the confirmatory cohort I) to evaluate previously reported observations and to establish, if possible, a panel of potential biomarkers that would support early PD diagnosis; and a second confirmatory phase II (employing the confirmatory cohort II) to replicate independently our findings. In the first step, we had identified biomarker discovery studies performed in late stage PD patients, which employed appropriate analytical method and were sufficiently powered to select CSF protein markers. To this end, we selected 15 protein markers–Aβ40, Aβ42, α-syn, p-Tau, t-Tau, OPN, HMGB1, NFL, IL-6, DJ-1, UCHL1, FLT3LG, MMP2, S100β and ApoA1 –for measurement in the CSF of confirmatory cohort I (comprising 80 early clinical PD patients and 80 controls) and subsequently evaluated their robustness to distinguish early clinical PD patients from controls. All selected protein markers participate in crucial pathways involved in PD pathogenesis and were previously considered as promising protein markers for late stage PD patients [13, 15, 21–44]. Through machine learning and univariate analysis, we aimed to identify protein biomarkers in CSF, which are robust and reproducible enough to utilize in the diagnosis of the early stages of PD.

## Material and methods

### Sample collection and patients

Early clinical PD patients and controls were recruited from the outpatient clinic at the Neurodegenerative Department of the University of Tübingen, Germany, and clinical data was collated (Table 1). The study was approved by the Ethics Committee of the Medical Faculty of the University of Tübingen (480/2015BO2). All participants provided written informed consent. PD was diagnosed according to the United Kingdom Brain Bank Society Criteria [45]. All patients were investigated by movement disorders specialists, to keep the risk of misdiagnosis at a minimum. Control individuals were assessed as having no neurological disease. Early clinical PD patients were chosen to represent a homogeneous cohort with very early disease state (mean disease duration = 1.89 years, median Hoehn and Yahr stage (H&Y) = 2, and median Unified Parkinson's disease rating scale III (UPDRS III) = 23) and to have the akinetic-rigid subtype of PD [46, 47]. We included only akinetic-rigid patients as there is increasing evidence that tremor-dominant and akinetic-rigid subtypes are the consequence of different pathopysiologies, to increase the probability to find (subtype-) specific results [48, 49]. CSF was collected by lumbar puncture in fasting patients according to standardized guidelines previously described in the literature [50]. To prevent blood contamination, CSF samples were tested for hemoglobin. Hemoglobin was determined by photometric Hb concentration measurements at 545 nm, conducted using ADVIA 1800 (Siemens Healthcare, Erlangen, Germany). In all measured samples hemoglobin concentration was <0.01 g/L. CSF samples free of blood were centrifuged (1600 g, 4°C, 15 min), frozen within 30–40 min after the puncture and stored at -80°C according to CSF collection and storage guidelines [51].

**Table 1:** Cohort summary.

Confirmatory Cohort I			Confirmatory Cohort II	
	PD	Controls	PD	Controls
**Individuals (n)**	80	80	30	30
**gender (male in % (m/f))**	51% (54/26)	49% (51/29)	63% (19/30)	43% (13/30)
**age (in years mean +/- SD)**	64.28 ± 9.8	62.74 ± 10.2	64.93 ± 9.1	59.27 ± 14.6
**Disease duration (in years mean)** **LEDD median (IQR)** **H&Y median (range 1–4)** **MMSE median (IQR)** **MoCA median (IQR)** **UPDRS III median (IQR)** **BDI (median IQR)**	2 ± 1.1 160 (353) 2 29 (3) 27 (4) 23 (15) 7 (5)	NA NA NA 30 (1) 28 (3) 0 (1) 2 (4)	2± 1.1 179 (324) 2 29 (2) 27 (2) 23 (15) 7 (4)	NA NA NA NA 29 (4) NA NA

A total of 220 individuals were included in the this study. Confirmatory cohort I comprised 80 early clinical PD patients and 80 controls. For confirmatory phase II, a total of 60 individuals were included, 30 early clinical PD patients and 30 controls. Although collected at the same institution, the cohorts were independent from each other with regard to recruitment time point and time of analysis. In more detail, samples of the second cohort were analyzed entirely independently from the samples of the first cohort, including a later time point and fit for purpose assays. Gender, age and disease duration were calculated for both groups and are presented below. H&Y = Hoehn and Yahr staging; UPDRS III = Unified Parkinson's Disease Rating Scale. NA = not available. IQR = interquartile range (Q3-Q1).

### Ligand binding assay measurement

Quantitative determination of selected markers was done by ELISA following manufacturer’s guidelines (Table 2) and validated fit-for-purpose as proposed by Jani et al [52].

**Table 2:** Analyte information.

Analyte	Diluiton	Limit of detection	Intra assay precison	Company
α-syn	1:1	0.37pg/mL	<15% CV	Analytik-Jena, Germany
DJ-1	8-fold	12.0 pg/mL	<10% CV	Meso Scale Discovery, USA
FLT3LG	8-fold	0.49 pg/mL	<10% CV	Meso Scale Discovery, USA
UCHL1	2-fold	0.31 ng/mL	<10% CV	Millipore, USA
MMP2	2-fold	200 pg/mL	<5.4% CV	Millipore, USA
S100β	2-fold	2.7 pg/mL	<4% CV	Millipore, USA
p-Tau	1:100	7.8 pg/mL	<15% CV	Fujirebio, Germany
t-Tau	1:100	7.8 pg/mL	<15% CV	Fujirebio, Germany
Aβ42	1:100	7.8 pg/mL	<15% CV	Fujirebio, Germany
Aβ40	1:100	7.8 pg/mL	<15% CV	Fujirebio, Germany
ApoA1	2-fold	0.7 ng/mL	<10% CV	Millipore, USA
HMGB1	1:1	2.5 ng/mL	<15% CV	IBL International, Germany
OPN	5-fold	5 ng/mL	<8% CV	IBL International, Germany
NFL	5-fold	100 ng/L	<10% CV	IBL International, Germany
IL-6	2-fold	1.56 pg/mL	<10% CV	IBL International, Germany

### Statistical methods

All analyses were conducted using R-Studio (2016; Version v1.0.136). Demographic and baseline characteristics of the cohorts were assessed using summary statistics. Differences in means between early clinical PD and Controls were assessed using Analysis of Variance (ANOVA); differences in proportions were assessed using chi-squared tests.

### Analysis of CSF protein data

Firstly, univariate association analyses comparing early clinical PD and controls were conducted for each biochemical marker using a Wilcoxon Signed-Rank Test. A False Discovery Rate (FDR) adjustment was applied to control the type I error within each sample type and sensitivity analyses were conducted to assess the potential effect of outliers.

Multivariate analyses were conducted using the CARET package in R. Data CSF markers were modelled independently. Initial pre-processing of the biomarker data included: centre and scale, removal of near-zero variance predictors, replacement of values below the limit of quantification (BLQ) with a value of half the lower limit of detection, and imputation of other missing values using multivariate K-Nearest Neighbour imputation (KNN). The final step before analysis was to randomise subjects into training and test sets in a ratio of 3:1.

Two multivariate methods were employed to build models predictive of disease status (PD vs. control): Elastic Net (Regularized Regression; GLMNET) and Gradient Boosted (GBM) Regression. Repeated 10-fold cross validation of the training set was used (for both methods) to give an indication of the accuracy of the resulting predictive models. The best Elastic Net and GBM models were chosen based on predictive accuracy. The selected models were then applied to the data in the test set and predictive probabilities were generated for each subject. Confusion matrices were produced and model fit was assessed using the following parameters: sensitivity, specificity and area under the Receiver Operating Characteristics (ROC) curve. For the confirmatory phase II, selected univariate and multivariate hypotheses generated above, were tested on an independent cohort of early clinical PD and controls. Univariate replication of the most promising CSF markers was assessed using a two group Wilcoxon Signed-Rank Test. Only one multivariate model, built by GBM using CSF markers, was tested for replication in the confirmatory cohort II. A selection of the most promising CSF markers was used to fit an example tree from the GBM model to the training set (early clinical PD and controls only) using CARET. Predictive probabilities were generated for each subject and the best cut-off from the training set was used to construct the confusion matrix, and calculate sensitivity, specificity and area under the Receiver Operating Characteristics (ROC) curve.

## Results

### Selection of potential CSF markers for the early diagnosis of PD

**Fig 1:**
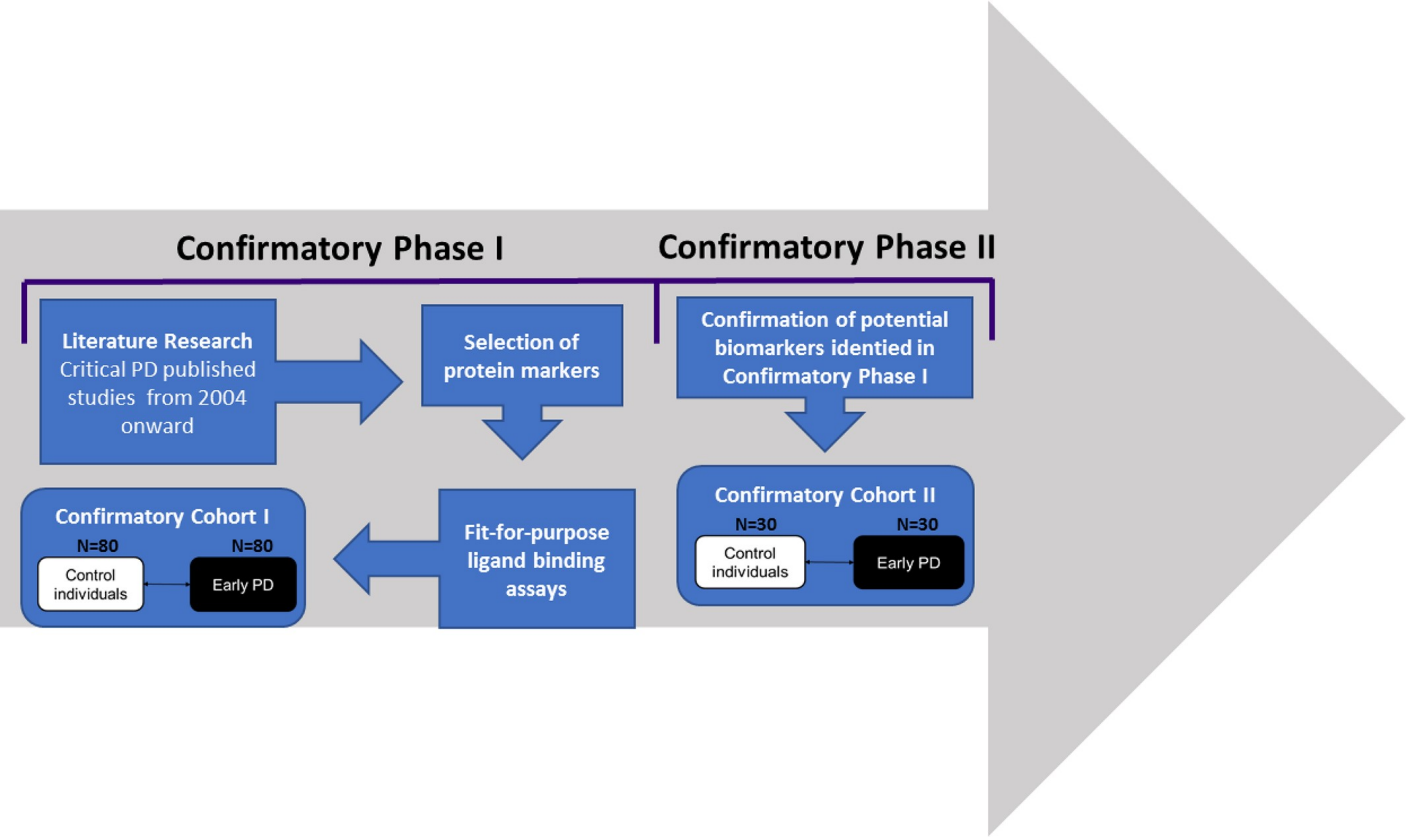
Biomarker study pipeline. Illustrative scheme representing study design. The study was divided in confirmatory phase I and confirmatory phase II. The confirmatory phase I comprised of literature research for selection of biochemical markers and analysis of selected markers in confirmatory cohort I. The confirmatory phase II comprised the validation of markers and models created in an independent cohort (confirmatory cohort II).

Our study was designed in two steps aligned to patient cohorts: confirmatory cohort I and confirmatory cohort II. Potential biomarkers or panels of biomarkers identified during the first confirmatory step, were taken forward to confirmatory step 2, where markers identified in step 1 should be confirmed in an independent cohort (confirmatory cohort II comprising 30 early clinical PD and 30 controls)(Fig 1)

The demographic characteristics of the cohorts are summarized in Table 1.

### Univariate analysis of selected markers

**Fig 2:**
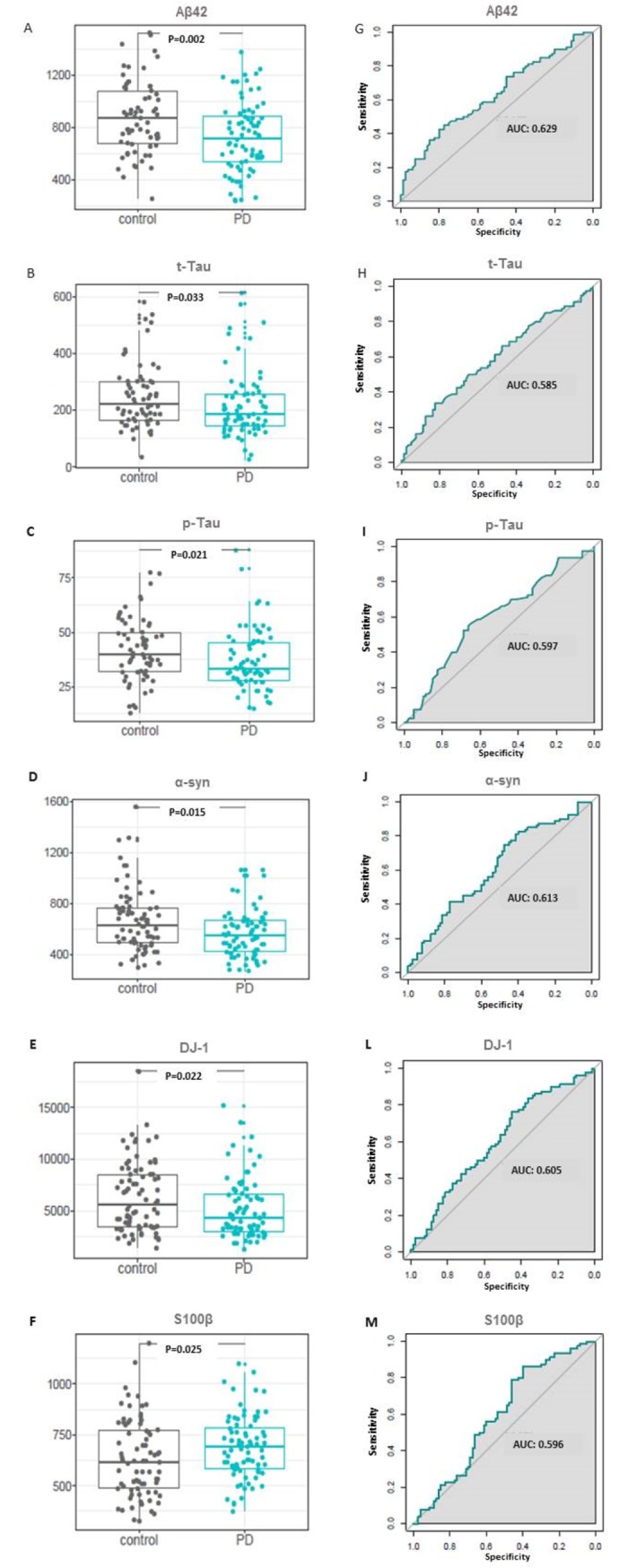
Univariate analysis of selected markers in confirmatory cohort I. (**A-F**) Boxplots of markers showing statistically significant changes in early clinical PD patients. Grey dots represent controls and blue dots represent early PD. P-values are calculated using the Wilcoxon signed-rank test. (**G-M**) Corresponding ROC-AUC analysis of significant markers.

Univariate analyses comparing early clinical PD and controls were conducted for each protein marker. From the 15 protein markers selected, 6 were found significantly dysregulated in early clinical PD. An overview of the results is shown in Fig 2. Aβ42, t-Tau, p-Tau, α-syn and DJ-1 were decreased in early clinical PD patients compared to the controls (unadjusted p = 0.002, p = 0.033, p = 0.021, p = 0.015, p = 0.022, p = 0.002, respectively), whereas S100β levels was increased in early clinical PD patients (p = 0.025) (Fig 2A–2F).

**Fig 3:**
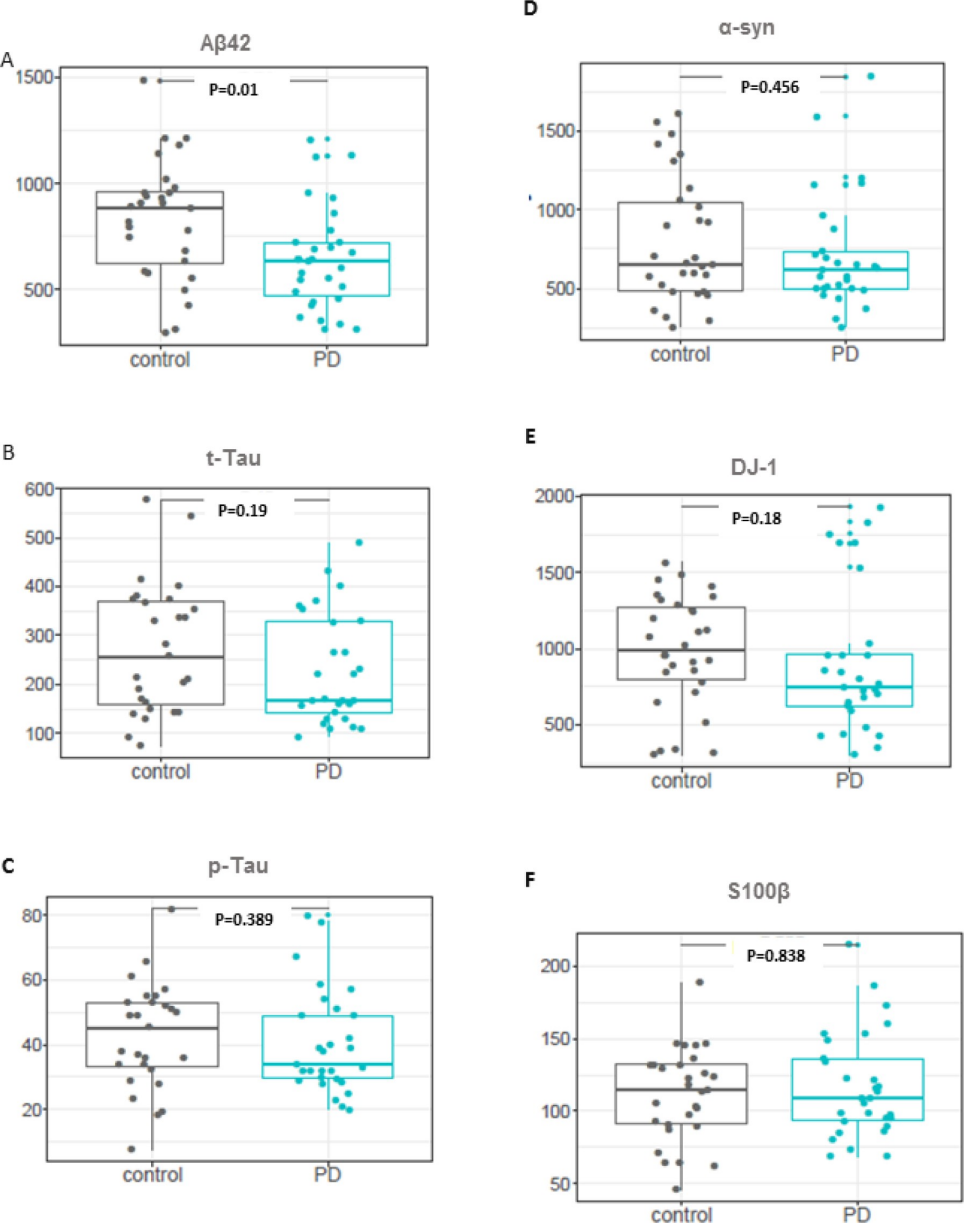
Univariate analysis of selected markers in confirmatory cohort II. (**A-F**) Boxplots of markers showing statistically significant changes in early clinical PD patients. P-values are calculated using the Wilcoxon signed-rank test. Grey dots represent controls and blue dots represent early PD.

To confirm these findings, we measured S100β, DJ-1, UCHL1, Aβ42, t-Tau, p-Tau and α-syn levels in the CSF of an independent, confirmatory cohort II, comprised of 30 early clinical PD patients and 30 controls. Of the 6 protein markers analysed, only Aβ42 levels were found to be significantly changed with levels decreased in early clinical PD patients compared to controls(Fig 3). Receiver Operating Characteristic (ROC) curve analysis was performed to determine the diagnostic accuracy of Aβ42, which presented 62% of area under the curve (AUC) (Fig 2 and S1 Fig).

### Multivariable analysis revealed a potential panel for the early diagnosis of PD

We employed machine learning approaches to investigate if the combination of markers could be used to accurately distinguish early clinical PD from controls. In order to identify robust panels, we employed two different machine leaning methods: Elastic Net (Regularized Regression; GLMNET) and Gradient Boosted (GBM) Regression.

**Fig 4:**
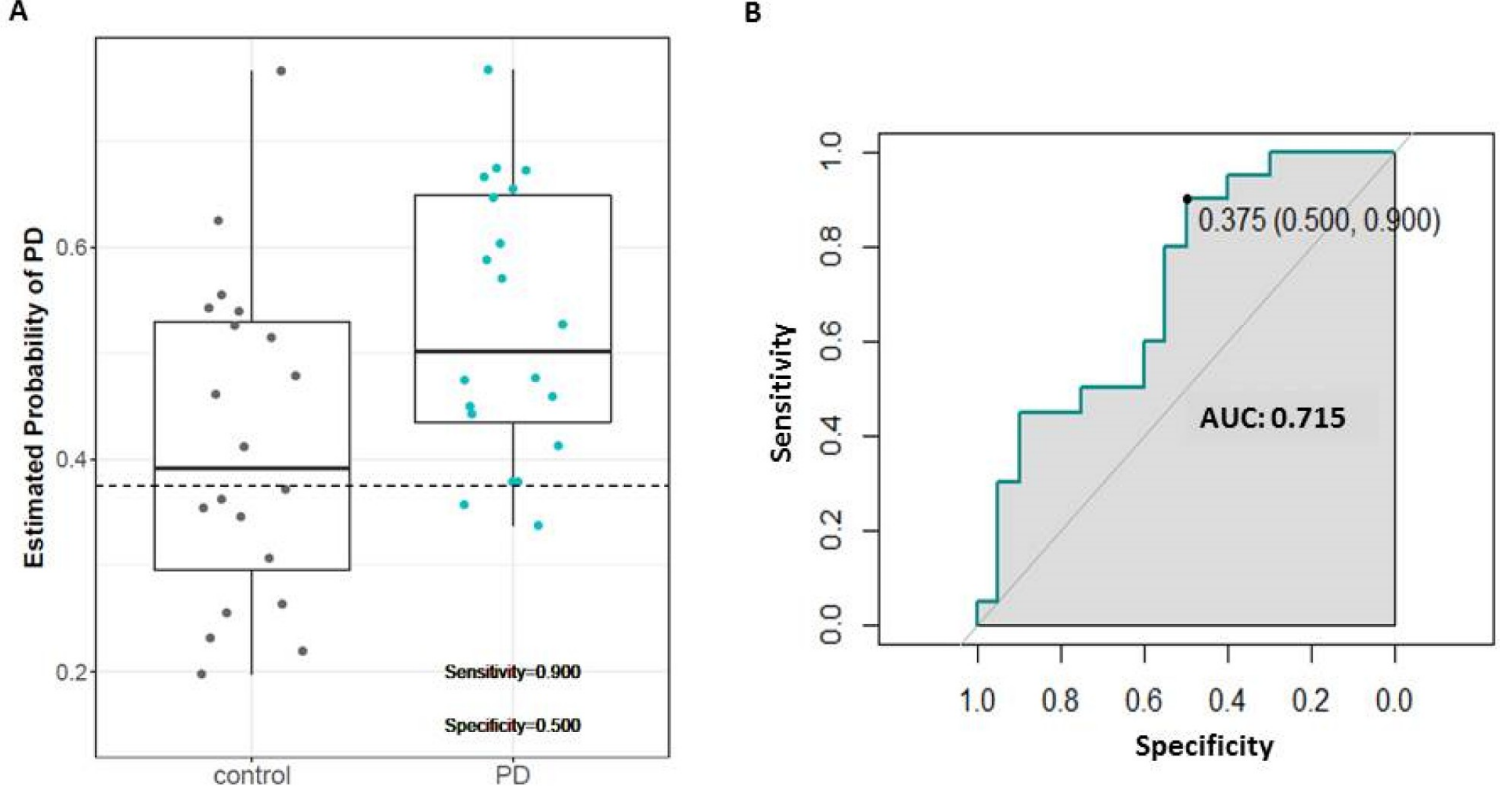
Diagnostic accuracy of potential model from Elastic Net Regression. **(A)** Predictive probabilities of PD from the most promising Elastic Net Model. The horizontal line corresponds to a predictive probability cut-off of 0.375 to classify PD and control. **(B)** Corresponding ROC curves, showing the AUC, optimal cut-off, sensitivity and specificity of the test.

Through Elastic Net algorithm, we first defined the ideal threshold to differentiate early clinical PD from controls. From the 15 markers analysed, 14 were taken forward and consequently we found a potential model comprising 16 variables: Aβ40, Aβ42, α-syn, p-Tau, t-Tau, OPN, NFL, IL-6, DJ-1, UCHL1, FLT3LG, MMP2, S100β, ApoA1, ratio Aβ40/Aβ42 and p-Tau/t-Tau. The model was able to distinguish early clinical PD from controls with 90% sensitivity, 50% specificity, 83% and 64% positive and negative predictive values, respectively. ROC analysis revealed AUC = 0.71 (Fig 4).

**Fig 5:**
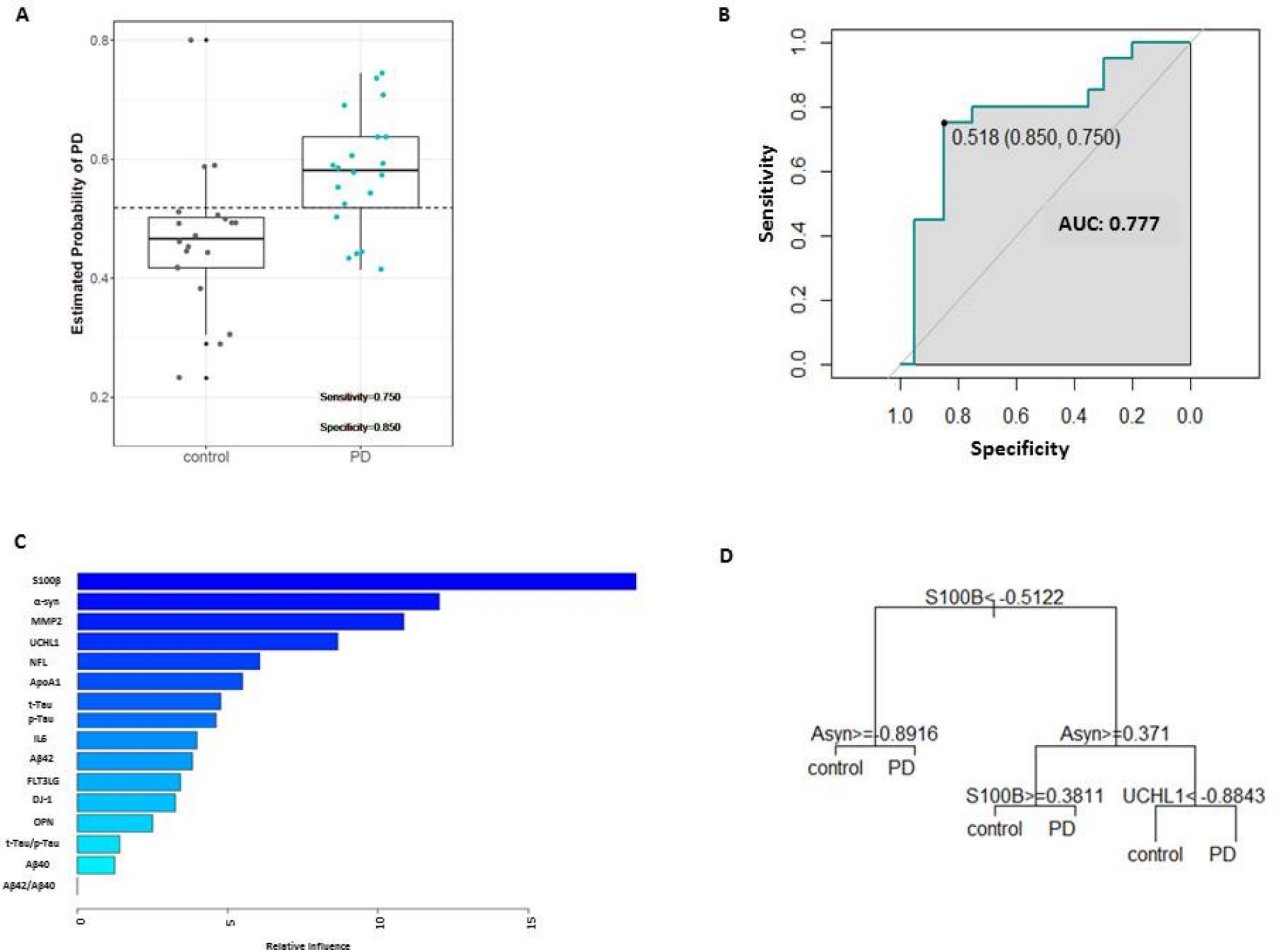
Diagnostic accuracy of gradient boosted model. **(A)** Predictive probabilities of PD from the most promising GBM. The horizontal line corresponds to a predictive probability cut-off of 0.518 to classify PD and control. **(B)** Corresponding ROC curves, showing the AUC, optimal cut-off, sensitivity and specificity of the test ROC curves of model created by gradient boosted regression. **(C)** Graph of most influencialfrequent variables. **(D)** Example of a decision tree.

In order to define a better classifier, we relied on the gradient boosted method. We employed a boosted decision tree algorithm to build an ensemble of 60 decision trees with an optimal interaction depth of four markers with 85% sensitivity, 75% specificity, 77% and 83% of positive and negative predictive values, respectively (Fig 5A). The analysis of diagnostic accuracy showed an AUC = 0.77 (Fig 5B). We used the ensemble of trees to rank the markers most frequent among all the trees and identified S100β, α-syn, MMP2 and UCHL1 as the most influential markers (Fig 5C). Among all trees presented in the model, the gradient boosted method suggests an interaction between S100β, α-syn and UCHL1, which seemed to be able to distinguish early clinical PD from controls (example tree shown in Fig 5D).

To validate the decision tree and associated protein markers, we applied the same analysis methods and cut-off values obtained with confirmatory cohort I in the confirmatory cohort II. The potential panel of markers were not reproducible in the independent cohort.

## Discussion

The discovery, development and validation of sensitive and specific biomarkers to support the early diagnosis of PD is currently an extensively debated topic. Although substantial progress in the discovery and development of low molecular weight and protein markers has been achieved, only a few candidates have been reported as promising biomarkers; however, none of them could be validated so far.

In this study, we aimed at confirming potential protein markers for PD as previously reported and assessing their applicability for early diagnosis during the first years of PD. Here, patients suffered from the disease for up to 36 months after first clinical diagnosis. To this end, we reviewed publicly available literature data from 2004 onward. We identified critical studies where biomarker discovery was conducted to identify PD potential biomarkers using well-characterized cohorts of sufficiently large numbers and in which the proposed biomarkers had a biological contextualization to PD [13, 15, 21, 23–31, 33, 34, 37, 53, 54]. These studies proposed protein markers differently affected mainly in late stage PD patients compared with age-matched controls. Therefore, we sought to confirm these protein markers in two cohorts of early clinical PD and to assess any potential diagnostic value. In total, 16 protein markers were selected, all of which are proposed to be associated with the major pathways of PD pathogenesis, such as protein aggregation (Aβ40, Aβ42, α-syn, p-Tau, t-Tau and NFL), inflammation (FLT3LG, MMP2, HMGB1, OPN and IL-6), oxidative stress (DJ-1 and S100β), lipid metabolism (ApoA1) and ubiquitin proteasome proteolysis (UCHL1) [13, 15, 21, 23–31, 33, 34, 37, 53, 54].

Given the importance of bioanalytical validity in the process of implementing biomarkers in the clinical setting, we pursued to the use of ligand binding assays to measure each marker. All commercial assays acquired as research-use-only were fit-for-purpose validated in order to ensure robustness of performance by means of improved analytics (S1 table) [52]. Each of the selected biochemical candidate markers was measured in CSF samples of a first confirmatory cohort comprised of 80 early clinical PD patients and 80 controls.

For statistical evaluation, we first employed an univariate analysis approach to evaluate which markers could be suitable to distinguish early clinical PD patients from controls. Power analysis had been conducted beforehand based on publicly available data in order to determine sample size for confirmatory cohort I. The univariate analysis revealed six protein candidates being significantly dysregulated in early clinical PD patients compared to controls: Aβ42, t-Tau, p-Tau, α-syn, DJ-1 and S100β. However, although these potential protein markers were found to be significantly different when comparing both groups, subsequent ROC-AUC analysis demonstrated limited robustness as diagnostic tools (Fig 2).

As many publications lack the replication of the findings in an independent cohort, we aimed at reinvestigating our observations in the confirmatory cohort II. The size of the cohort was based on power analysis based on data from confirmatory cohort I. Furthermore, as with confirmatory cohort I, the same randomization procedure was used for sample selection from the available much larger patient sample base; the bioanalytical and statistical procedures were identical to those applied in step 1.

The analysis of the data showed that of the 6 protein markers significantly dysregulated in confirmatory cohort I, only Aβ42 levels were confirmed as being significantly decreased in the confirmatory cohort II when compared to controls using univariate statistics (Fig 3). Decreased Aβ42 levels in CSF samples from PD patients had been reported recently, suggesting it may be a reliable candidate [55]. However, in this study, ROC analysis showed that this potential marker was not suitable for diagnostic purposes (Fig 2 and S1 Fig).

Classical statistical methods are powerful tools but recently have been complemented by emerging new tools, such as machine learning approaches, which can manage large numbers of variables and identify potential signatures based on correlated multivariate analysis. Furthermore, machine learning has the advantage of integrating multiple variables and clinical endpoints by using data mining and generating predictive algorithms to provide a meaningful representation of the relationship across the data. In line with that, we reanalyzed our data by using two different machine learning methods. We first used Elastic Net, an algorithm that identifies linear relations within the dataset, to find a model with the best combination of variables and the highest predictive values. Our analysis revealed a model with 16 variables (Aβ40, Aβ42, α-syn, p-Tau, t-Tau, OPN, NFL, IL-6, DJ-1, UCHL1, FLT3LG, MMP2, S100β, ApoA1, ratio of Aβ40/Aβ42 and ratio of p-Tau/t-Tau) able to distinguish early clinical PD from controls with 71% of diagnostic accuracy (AUC-ROC). Although high diagnostic sensitivity (90%) was achieved, diagnostic specificity (50%) was low suggesting lack of predictive value (Fig 4). When considering the biological relevance of the model, we observed that half of variables included in the model are associated with protein aggregation, one of the most important pathways of PD pathogenesis.

To improve predictivity and to better understand the relationship among the variables measured, we also used Gradient Boosted (GBM) Classification to define a model with higher predictivity and better interpretation. Through this method, we were able to identify a model comprising an ensemble of 60 decision trees with 85% diagnostic sensitivity, 75% diagnostic specificity, and 77% AUC-ROC (Fig 5A and 5B). We examined which markers impacted on the development of the model and found S100β, α-syn, MMP2 and UCHL1 as the most important markers to distinguish early clinical PD from controls (Fig 5C). Interestingly, this model aligned with findings published in the literature, where α-syn is characterized as the hallmark protein of PD, closely involved in the progression of neuronal degeneration and subsequent motor impairments, while S100β has been considered a possible marker for the accompanying neurodegeneration [56, 57]. Due to the novelty of this approach in the PD arena, we also aimed at confirming the model in confirmatory cohort II. When reproducing the model in this cohort, the decision tree could not be confirmed. Among potential reasons for this lack of reproducibility are (1) the less homogenous population included in the confirmatory cohort II and (2) the inherited, and previously documented, diagnostic limitation of the markers used in the model (3) imbalances in baseline characteristic between disease groups may have impacted the power of the replication study. Whereas the genders in confirmatory cohort I are equally distributed (controls 49% males, patients 51% males), the genders in the control group in cohort II are dominated by females (controls 43% males) whereas the patient group is dominated by males (63% males) (Table 1). This imbalance is also reflected in the distribution of DJ-1, S100β, t-Tau and p-Tau () (Table 1, S2 Fig).

Overall, in contrast to our expectations from recent literature data, we could only identify changes to Aβ42 in line with an earlier report [55]. Changes of a-syn, the hallmark of PD, could not be reproducibly detected in our two cohorts even with stringent experimental precautions. In a previous review, Mollenhauer et al. discussed the reliability of assays for the detection of a-syn in CSF in general as well as the desirable study design and CSF sampling conditions (ideally to be standardized between the analytical labs) [20]. Besides the pre-analytical handling of the samples, she also discussed whether post-translational modifications of a-syn (phosphorylation, ubiquitination, truncation, truncation and oxidization) and the various species of a-syn (monomeric, oligomeric, translationally modified) could be at least some of the reasons for the high variability and lack of reproducibility of reported findings between different groups and even–as in our case—even in the same laboratory. To this end, efforts of 18 different laboratories collaborating in the EU-BIOMARKAPD project exploring alternative approaches, essential requirements and standardization procedures are ongoing in the hope of finally finding ways for biomarker identification. Taking these proposals into account, we can state that our investigations have been performed in line with many of the proposed requirements. Thus, our findings rather raise further questions around the suitability of CSF as source of protein markers for PD, the potentially still insufficient clinical differentiation of PD subtypes, the need for further implementation of standardized experimental procedures and also the optimziation of statistical evaluations.

To our knowledge, this is the first study which evaluated the performance of literature-based protein markers proposed for late stage PD in early clinical PD patients in line with previously proposed requirements from the neuroscience arena [19]. Our data shows that univariate analysis of CSF protein analysis performed for late stage PD may have limited value in early clinical PD diagnosis. Aβ42 levels only could be shown to be reproducible in both of our cohorts. However, whether changes of Aβ42 levels may contribute to the differentiation of PD from other neurodegenerative diseases remains a question. Alternative data analysis using a multivariate approaches such as the machine learning methods employed here might be of help in the development of a PD diagnostic algorithm.

## Conclusions

In this study, we selected CSF proteins proposed as potential biomarkers for PD and assessed their applicability in two sets of samples from patients in early clinical stages of PD. We optimized and employed appropriate experimental conditions to measure and analyse these markers in the CSF the patients and controls. We demonstrate here that the currently proposed protein CSF markers for PD diagnosis, as identified in late stage PD cohorts, lack robustness and reproducibility when applied in the early clinical stages of akinetic-rigid PD. Thus, further efforts as the EU-BIOMARKAPD project might support the development of potential protein CSF biomarkers for diagnosis or disease monitoring in early clinical PD.

## Supporting information

S1 Fig Diagnostic accuracy of Aβ42. **(A)** Predictive probabilities of PD from a univariate model of Aβ42. The horizontal line corresponds to a predictive probability cut-off of 0.559 to classify PD and control. **(B)** Corresponding ROC curves, showing the AUC, optimal cut-off, sensitivity and specificity of the test.
(TIF)

S2 Fig Assessment of potential confounding factors. Figures (A-F) show the relationship between age, gender and protein levels in the two groups.
(TIF)

S1 Table Summary of the validation criteria. (XLSX)
