# Supplementary figures and images for: Evaluation of cerebrospinal fluid proteins as potential biomarkers for early stage Parkinson’s disease diagnosis
Source: PLoS One. 2018 Nov 1;13(11):e0206536. doi: 10.1371/journal.pone.0206536 (PMC6211693; doi:10.1371/journal.pone.0206536)

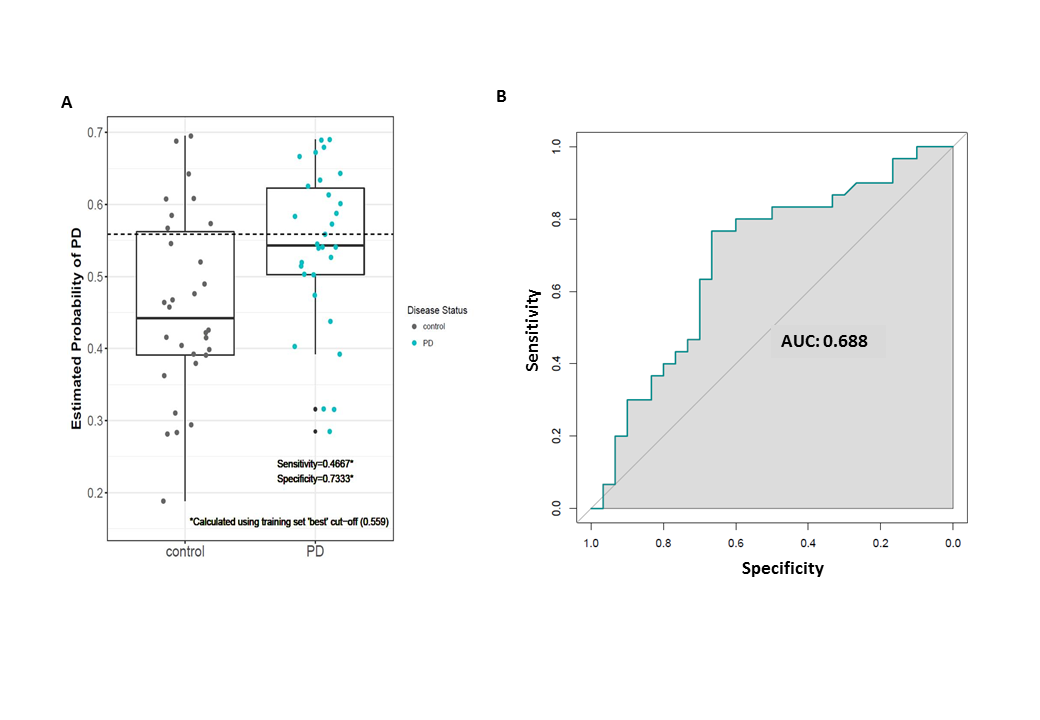

Supplement: S1 Fig — (A) Predictive probabilities of PD from a univariate model of Aβ42. The horizontal line corresponds to a predictive probability cut-off of 0.559 to classify PD and control. (B) Corresponding ROC curves, showing the AUC, optimal cut-off, sensitivity and specificity of the test. (TIF) [file pone.0206536.s001.tif]

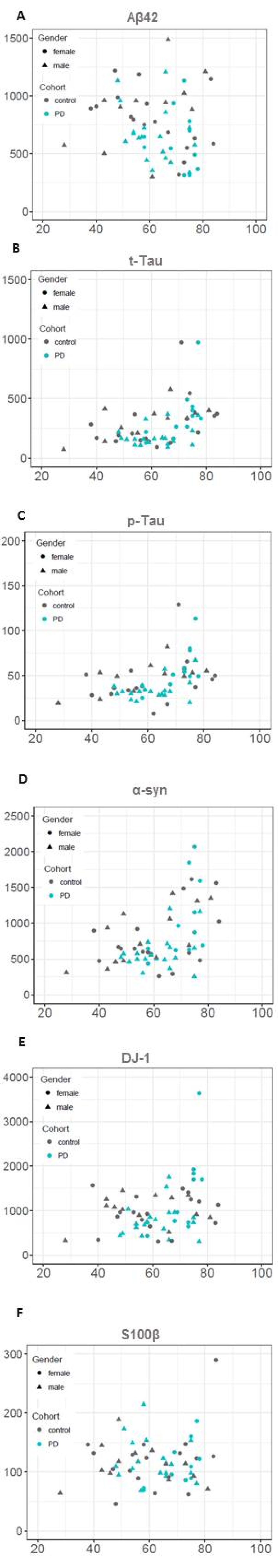

Supplement: S2 Fig — Figures (A-F) show the relationship between age, gender and protein levels in the two groups. (TIF) [file pone.0206536.s002.tif]
